# Automated home-cage monitoring as a potential measure of sickness behaviors and pain-like behaviors in LPS-treated mice

**DOI:** 10.1371/journal.pone.0256706

**Published:** 2021-08-27

**Authors:** Hasriadi, Peththa Wadu Dasuni Wasana, Opa Vajragupta, Pornchai Rojsitthisak, Pasarapa Towiwat

**Affiliations:** 1 Faculty of Pharmaceutical Sciences, Pharmaceutical Sciences and Technology Program, Chulalongkorn University, Bangkok, Thailand; 2 Faculty of Allied Health Sciences, Department of Pharmacy, University of Ruhuna, Galle, Sri Lanka; 3 Faculty of Pharmaceutical Sciences, Research Affairs, Chulalongkorn University, Bangkok, Thailand; 4 Natural Products for Ageing and Chronic Diseases Research Unit, Chulalongkorn University, Bangkok, Thailand; 5 Faculty of Pharmaceutical Sciences, Department of Food and Pharmaceutical Chemistry, Chulalongkorn University, Bangkok, Thailand; 6 Faculty of Pharmaceutical Sciences, Department of Pharmacology and Physiology, Chulalongkorn University, Bangkok, Thailand; University of Southern California, UNITED STATES

## Abstract

The use of endotoxin, such as lipopolysaccharide (LPS) as a model of sickness behavior, has attracted recent attention. To objectively investigate sickness behavior along with its pain-like behaviors in LPS-treated mice, the behavioral measurement requires accurate methods, which reflects clinical relevance. While reflexive pain response tests have been used for decades for pain assessment, its accuracy and clinical relevance remain problematic. Hence, we used automated home-cage monitoring LABORAS to evaluate spontaneous locomotive behaviors in LPS-induced mice. LPS-treated mice displayed sickness behaviors including pain-like behaviors in automated home-cage monitoring characterized by decreased mobile behaviors (climbing, locomotion, rearing) and increased immobility compared to that of the control group in both short- and long-term locomotive assessments. Here, in short-term measurement, both in the open-field test and automated home-cage monitoring, mice demonstrated impaired locomotive behaviors. We also assessed 24 h long-term locomotor activity in the home-cage system, which profiled the diurnal behaviors of LPS-stimulated mice. The results demonstrated significant behavioral impairment in LPS-stimulated mice compared to the control mice in both light and dark phases. However, the difference is more evident in the dark phase compared to the light phase owing to the nocturnal activity of mice. In addition, the administration of indomethacin as a pharmacological intervention improved sickness behaviors in the open-field test as well as automated home-cage monitoring, confirming that automated home-cage monitoring could be potentially useful in pharmacological screening. Together, our results demonstrate that automated home-cage monitoring could be a feasible alternative to conventional methods, such as the open-field test and combining several behavioral assessments may provide a better understanding of sickness behavior and pain-like behaviors in LPS-treated mice.

## Introduction

Inflammatory pain is an excessive immune response which sensitizes the peripheral and central nerves leading to a robust transmission of pain [1]. In pathological conditions associated with inflammatory pain, such as arthritis, fibromyalgia, and neuropathy, pain is persistent and decreases the affective function, physical activity, and well-being [1–4]. In the management of pain, the effectiveness of most of the therapies remains inadequate. Hence, it is essential to consider new drugs that could overcome the limitations of the existing therapies [5]. However, a low rate of translation from preclinical to clinical studies is likely a main factor contributing to the failure to develop new drugs. Only 11% of the investigated compounds in clinical trials is eventually approved to be used as drugs [6]. Many drugs exhibit efficacy in animal models of pain have failed in clinical trials due to several reasons including diverse pathophysiology of pain in rodents and humans, limited well-established molecular targets, and limitations of preclinical design and pain assessment [5,7].

In the past few years, endotoxins such as lipopolysaccharide (LPS) was used as a model to study sickness behavior. Sickness behavior is an immune response, which manifested by flu-like symptoms, fatigue, sleep disturbance, reduced physical activity, mild cognitive disorder, social withdrawal, anorexia, pyrexia, and pain [8–13]. Some of the behavioral signs of sickness behavior overlap with pain-like behaviors, such as reduction of locomotor activity (physical disability), weight loss, and appetite [14–16]. At the mechanistic levels both sickness and pain-like behaviors are associated with increased pro-inflammatory mediators [8,17]. Therefore, the use of endotoxin has been recently introduced as a model for the study of inflammatory pain in both animals and humans [18–25]. The administration of LPS produces a significant expression of proinflammatory mediators, like cytokines, chemokines, cyclooxygenase-2 (COX-2), and nitric oxide through activation of toll-like receptor 4 (TLR4) via modulating immune cells [26–28]. These inflammatory mediators play a pivotal role in the pathophysiology of many chronic pain conditions and act as a pronociceptive agent via sensitization of peripheral and central nerves [29–31]. Despite the ability to modulate the immune response, LPS directly activates nociceptors via transient receptor potential channels (TRP channels), such as the transient receptor potential cation channel subfamily V member 1 (TRPV1) [32] and the transient receptor potential ankyrin 1 (TRPA1) [33]. Regarding its behavioral effect, the administration of LPS resulted in increased sickness behaviors, pain hypersensitivity and spontaneous pain in rodents and humans [13,24,34–36]. Moreover, the use of endotoxin has been validated and used as a potential model for evaluating analgesic candidate drugs in clinical trials [37].

Currently, most of the pain studies are mainly provide information only on reflexive pain behaviors, with a lack of data on spontaneous pain behaviors [38]. These reflexive pain assessments are labor and time consuming and only measure a few behaviors [38–40]. Reflexive pain measurements such as thermal and mechanical hypersensitivities use only the sensory component to measure the pain response and do not accurately reflect the clinical features of pain [39]. Moreover, the existence of thermal and mechanical hypersensitivities in chronic pain patients is only 38% and 64%, respectively, while spontaneous pain can be observed in nearly all patients with chronic pain [41]. In addition, as shown in many studies, reflexive pain behaviors possess a high probability of giving false-positive/negative results due to several factors, like over handling of animal, restraining of animal (unnatural), the variability of human-animal interaction, and experimenter bias [39,41–44].

The use of non-reflexive assessments has recently attracted the attention of many researchers in the field of pain. Non-reflexive pain behaviors provide more clinical relevance associated with depressive behaviors, facial expression, motivational state, and functional disability (locomotor activity) [45,46]. In an attempt to overcome the limitations of reflexive pain, several methods have been devised including, nesting behavior, burrowing behavior, grimace scale, operant behavior, wheel-running activity, open-field test, gait analysis, and home-cage monitoring [40,46]. Most of these non-reflexive assessments consider the locomotor activity as a readout of behavioral pain. Specifically, measuring locomotor activity driven by exploratory behaviors by using the open-field test, a classical model of locomotor activity, is often used as a behavioral phenotype to measure pain state and test the potency of analgesic agents [46–51]. However, the assessment is often conducted in a short duration and during the biological night of mice (daytime). Therefore, in this study, we aimed to investigate the home-cage behaviors (spontaneous behaviors) associated with sickness behaviors induced by LPS in mice. The use of automated home-cage monitoring could limit human intervention and provides a home-like environment to mice. This approach can also precisely capture behavioral phenotype in seconds-to minutes, hours and days [52].

Therefore, in the present study, we assessed LPS-induced sickness behavior in both open-field and automated home-cage monitoring Laboratory Animal Behavior Observation Registration and Analysis System (LABORAS). The pharmacological intervention using indomethacin was applied in LPS-induced mice to validate the model. Hence, this study may help to add to the toolbox a technique that bridges the gap between animal and human pain research.

## Materials and methods

### Animals

Male ICR mice weighing 18–25 g were purchased from Nomura International (Bangkok, Thailand) and housed at the animal facility of Faculty of Pharmaceutical Sciences, Chulalongkorn University. Mice were given with food and water *ad libitum* and a 12/12 h light/dark cycle. Mice were maintained according to the guidelines, and experimental procedures, which were approved by the Committee of Institutional Animal Care and Use Committee (IACUC). Approval for the study was granted by the IACUC of the Faculty of Pharmaceutical Sciences, Chulalongkorn University, Thailand (protocol number: 1933006 & 2033004). At the end of the experiments, mice were euthanized with CO_2_ euthanasia. Efforts were made to minimize potential suffering and stress of the animals.

### Study design and experimental timeline

**Fig 1:**
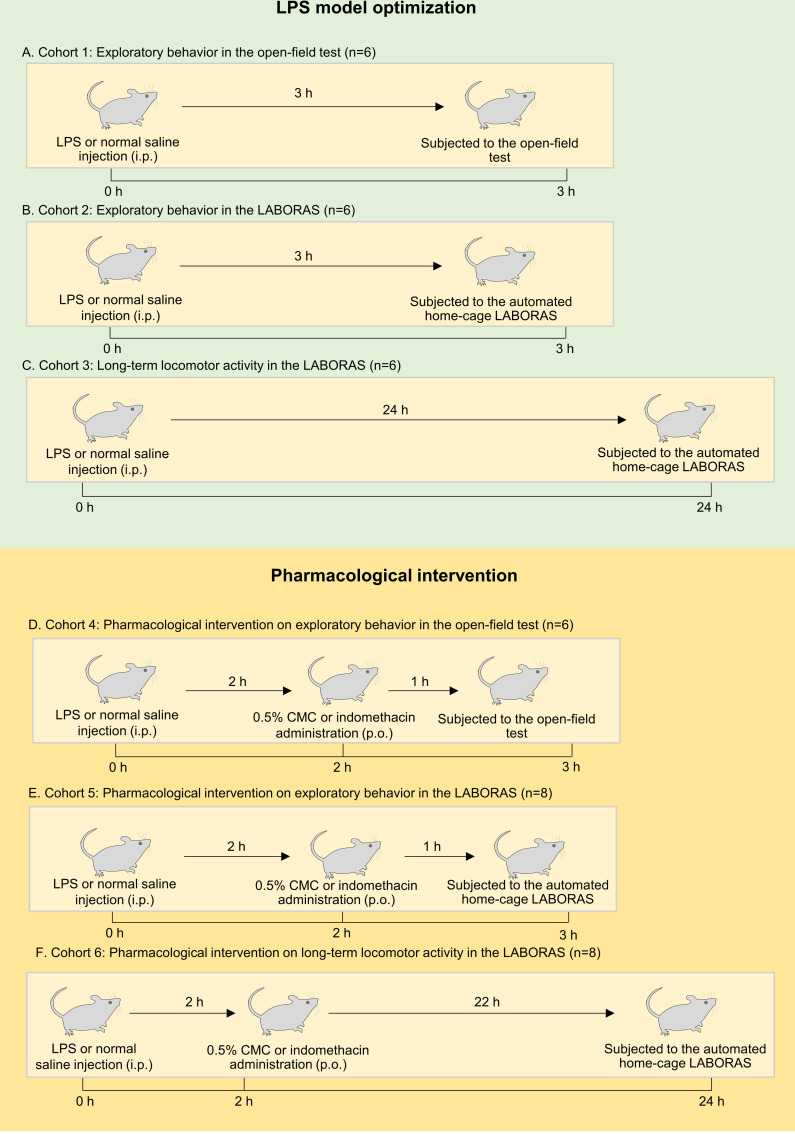
Experimental timeline for different cohorts of mice. Cohort 1: Mice subjected to the open-field test to assess the effect of LPS on exploratory behaviors (A). Cohort 2: Mice subjected to automated home-cage LABORAS to measure exploratory behaviors, Cohort 3: Mice subjected to automated home-cage LABORAS to measure the effect of LPS on long-term locomotor activity (C). Cohort 4, 5, 6: Mice used for pharmacological intervention using indomethacin in the open-field test (D), automated home-cage monitoring for short- and long terms (E and F, respectively).

The study design and experimental timeline are presented in **Fig 1**. Six cohorts of mice were used in the present study. Behavioral assessments including short-, and long-term locomotor activity were carried out in male ICR mice (6–8-week-old). Three cohorts (cohort 1–3) of mice compromising vehicle- and LPS-treated groups were used to assess the effect of LPS challenge on exploratory behaviors separately in the open-field test and LABORAS, and long-term locomotor activity in LABORAS. The results of cohort 1–3 were used to determine the effect of LPS and appropriate time to administer the standard drug for pharmacological intervention **(Fig 1A–1C)**. Subsequently, three cohorts of mice (cohort 4–6) containing vehicle-, LPS-, and LPS+indomethacin-treated groups were used to evaluate the effect of pharmacological intervention. Cohort 4–6 were subjected to evaluate the exploratory behavior in open-field, exploratory behaviors in LABORAS and long-term locomotor activity in LABORAS, respectively. For the pharmacological intervention of exploratory behaviors in the open-field and LABORAS, indomethacin was administered 2 h post-LPS challenge, and the behaviors were measured 1 hour later **(Fig 1D and 1E)**. In long-term locomotor activity, mice were injected with LPS and placed into the LABORAS cage. After 2 h, mice were taken out, administered indomethacin and behaviors were measured continually for 24 h **(Fig 1F)**.

### LPS-induced sickness behavior

Mice were injected intraperitoneally (i.p.) with 1 mg/kg of lipopolysaccharide (LPS; Sigma, St. Louis, MO, USA)) to produce sickness behavior as well as pain-like behavior, which was selected based on the previous studies [53,54]. The behaviors were then evaluated using the open-field test, a classical locomotor activity test, and automated home-cage monitoring.

### Drug administration

Indomethacin (Sigma, St. Louis, MO, USA) was suspended in 0.5% carboxylmethycellulose (CMC) in normal saline and then administered orally (10 mg/kg) to mice 2 h after LPS induction.

### Behavioral testing

In the present study, classical locomotor activity test (open-field test) and automated behavioral system LABORAS were used to evaluate sickness behavior together with pain-like behavior in LPS-induced mice. Short term-locomotor activity both in the open-field and automated home-cage was assessed in the daytime (light phase) between 8.00–15.00 h. For long-term locomotor activity in automated home-cage monitoring, the test was conducted in the nighttime (dark phase) 18.00–17.59 h. Since mice are nocturnally active, the behaviors were evaluated for 24 h considering both the dark and light phases. On the day of the experiment, mice were acclimatized to the experimental room for 1 h before testing.

#### Open-field test

The open-field test was used to assess locomotor activity associated with sickness behaviors. Before each trial, the arena was carefully cleaned with isopropyl alcohol to eliminate olfactory cues from the previous test, and the mice were then placed in a black open-field box (50 × 50 × 50 cm). The movement of the mice was recorded by a video camera directed on the open-field box. The data, including the distance traveled, speed, and immobility were analyzed with a VideoMOT2 (TSE Systems, Bad Homburg, Germany).

#### Automated home-cage monitoring LABORAS

Sickness behaviors were analyzed using the automated home-cage monitoring LABORAS (Metris B.V., Hoofddorp, Netherlands). In this approach, short-term locomotor activity driven by exploratory behaviors and long-term locomotor activity were assessed for 5 min and 24 h, respectively. Behavioral measures of mice, including climbing, immobility, locomotion, and rearing, were illustrated in duration and frequency. The behavioral duration was the time spent by mice in a particular behavior, whereas behavioral frequency was how often a behavior occurred in each time. Several other behaviors such as locomotive characteristics, food, and water intake, and body weight changes were additionally monitored. Undefined behaviors obtained were excluded from analysis.

- **Climbing**: defined as the position of the mouse in hanging or climbing on the cage
- **Locomotion**: the mouse is jumping, walking, and running
- **Immobility**: defined as a resting state of the mouse or an absence of movement, such as sitting or lying position
- **Rearing**: the mouse put its weight on its hind paw and standing upright where its forelimbs do not touch the floor of the cage
- **Position distribution**: the position distribution of the mouse throughout the cage
- **Distance traveled:** the total distance traveled by the mouse during the experiment
- **Average speed**: the average velocity of the mouse during the locomotive periods (distance traveled/duration of locomotive behaviors)
- **Food and water intakes**: the total amount of food and water consumed by the mouse for 24 h and expressed in gram and mL, respectively. Along with the food and water intake, mouse’s body weight was also monitored before and after the experiment

### Statistical analysis

The data and statistical analysis were performed using GraphPad Prism 9.1 (GraphPad, San Diego, CA). All values are reported as mean ± SEM. The level of statistical significance for all tests was set at *p*<0.05. t-test, one-way ANOVA, with Bonferonni *post-hoc* test, were used to compare groups.

## Results

### LPS-induced sickness behaviors, exploratory behavior-driven locomotor activity in the open-field test

**Fig 2:**
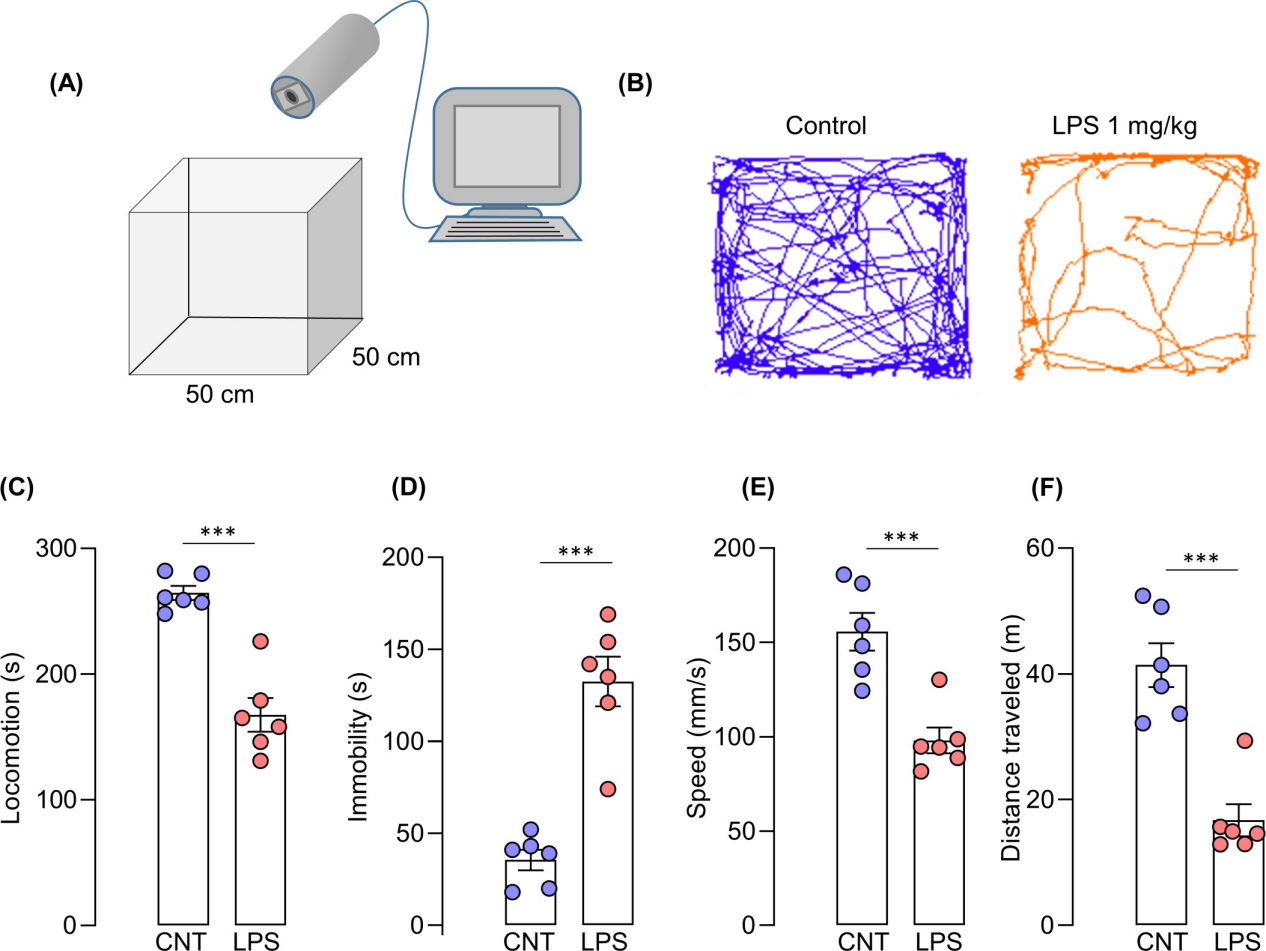
Locomotor activity deficits in LPS-induced mice. (A) Experimental setup, (B) travel traces of mice in the arena, (C) locomotion (s), (D) immobility (s), (E) speed (mm/s), (F) distance traveled (m). Unpaired t-test, N = 6 mice per group; ***p < 0.001 compared with the control group.

To determine exploratory behaviors of mice with the LPS-induced sickness behavior, we subjected mice to the open-field test to assess locomotor activity. The test was performed 3 h after administration of LPS. Mice treated with LPS displayed deficits in locomotor activity compared to the vehicle-treated group. The reduction of locomotor activity in the LPS group was indicated by significant reduction in locomotion (s) (t _(10)_ = 6.660, p < 0.001), speed (cm/s) (t _(10)_ = 4.735, p = 0.0008), distance traveled (t _(10)_ = 5.701, p = 0.0002), and increased immobility (t _(10)_ = 6.660, p < 0.001) **(Fig 2)**.

### LPS-induced sickness behaviors, exploratory behavior-driven locomotor activity in the automated home-cage monitoring

**Fig 3:**
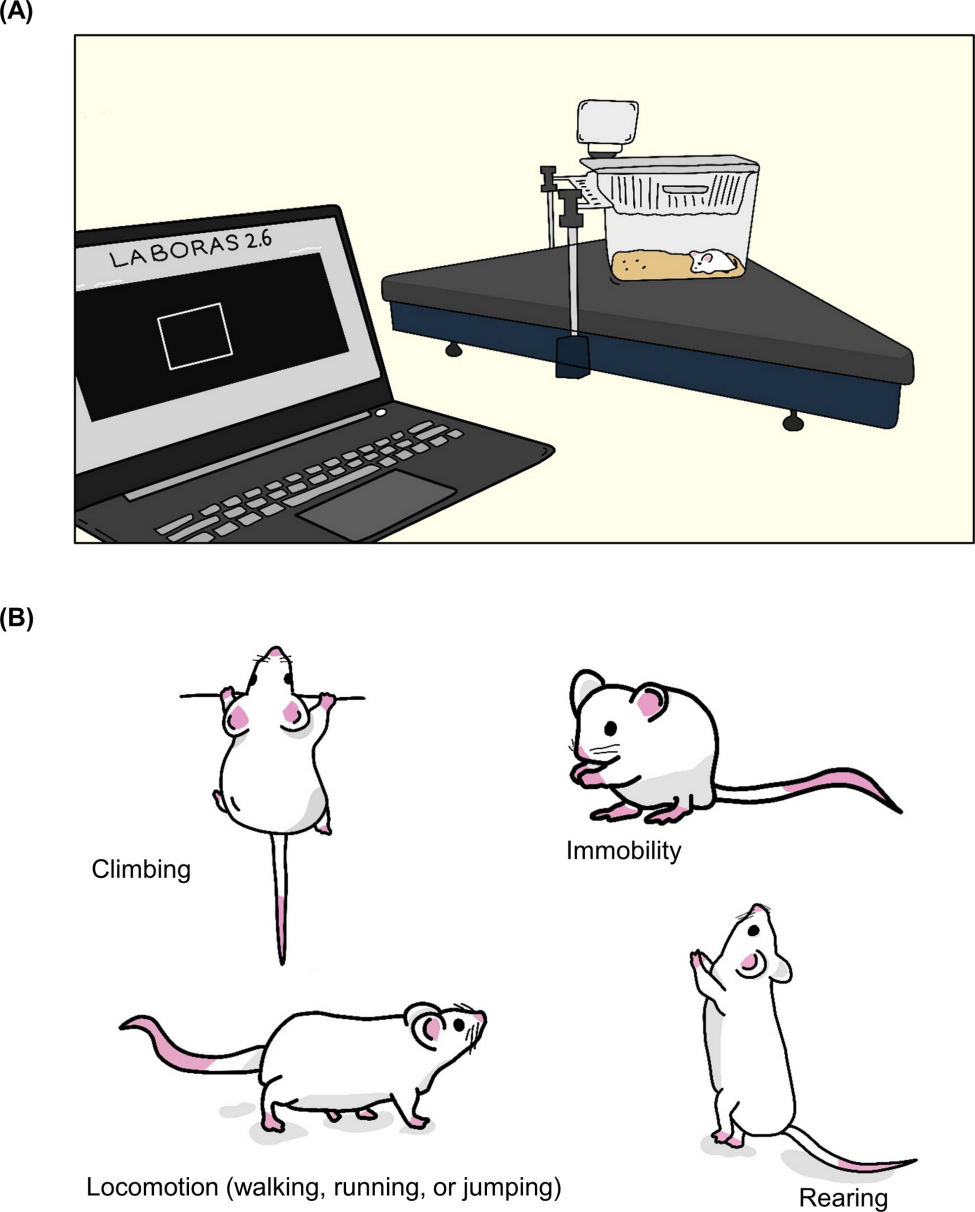
Schematic representation of the experimental setup. (A) The LABORAS consists of cages connected to the LABORAS control unit (LCU) and computer. (B)The behaviors read by the automated home-cage LABORAS system.

**Fig 4:**
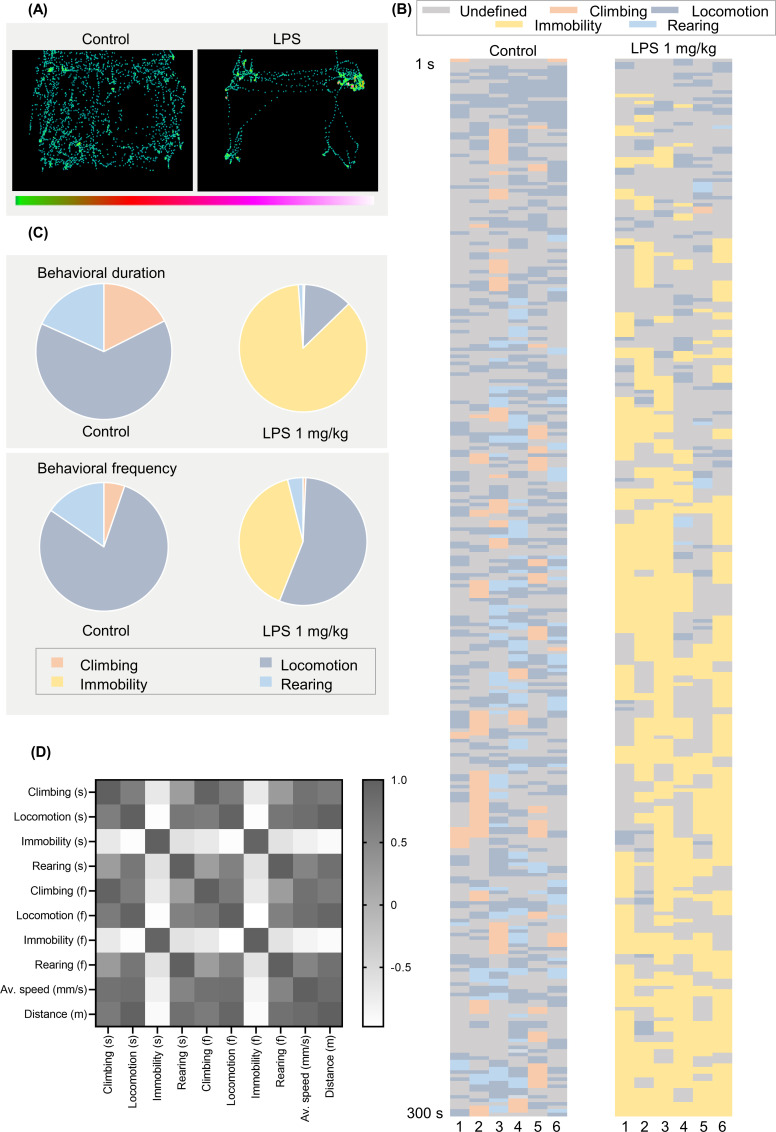
Automated home-cage behavioral profiling of LPS-induced mice in short–term locomotor activity (exploratory behavior). (A) position distribution, (B) ethogram, (C) percentage of behavioral composition (duration and frequency), derived by either duration or frequency of each behavior/total duration or frequency of identified behaviors, (D) correlation matrix of each behavior.

To gain further insights into the effect of LPS on exploratory behaviors, mice were subjected to automated home-cage monitoring. Three hours after the LPS challenge, exploratory behavior was measured for 5 minutes. We quantified different behaviors: climbing, locomotion, immobility, rearing, average speed, and distance traveled. Each mouse was placed individually into a cage of LABORAS, and the behaviors were recorded (**Fig 3**). To qualitatively demonstrate detailed behaviors of mice, the behaviors are indicated by position distribution and ethogram (**Fig 4A and 4B**). Position distribution allows us to see the position and track of the mouse in the cage, while the ethogram provides details of the behaviors in each second. In the ethogram, behaviors are visualized with different colors to indicate the difference in behaviors between the vehicle- and LPS-treated groups. Ethogram was drawn in each second and determined in accordance with the highest home-cage behavior of mice per second. As shown in **Fig 4A**, the position of the vehicle-treated mice in the cage demonstrates the highest extent of locomotion throughout the cage. In addition, the ethogram indicates that the vehicle control group predominantly exhibited mobile behaviors, whereas the LPS group mostly exhibited immobility (**Fig 4B**).

**Fig 5:**
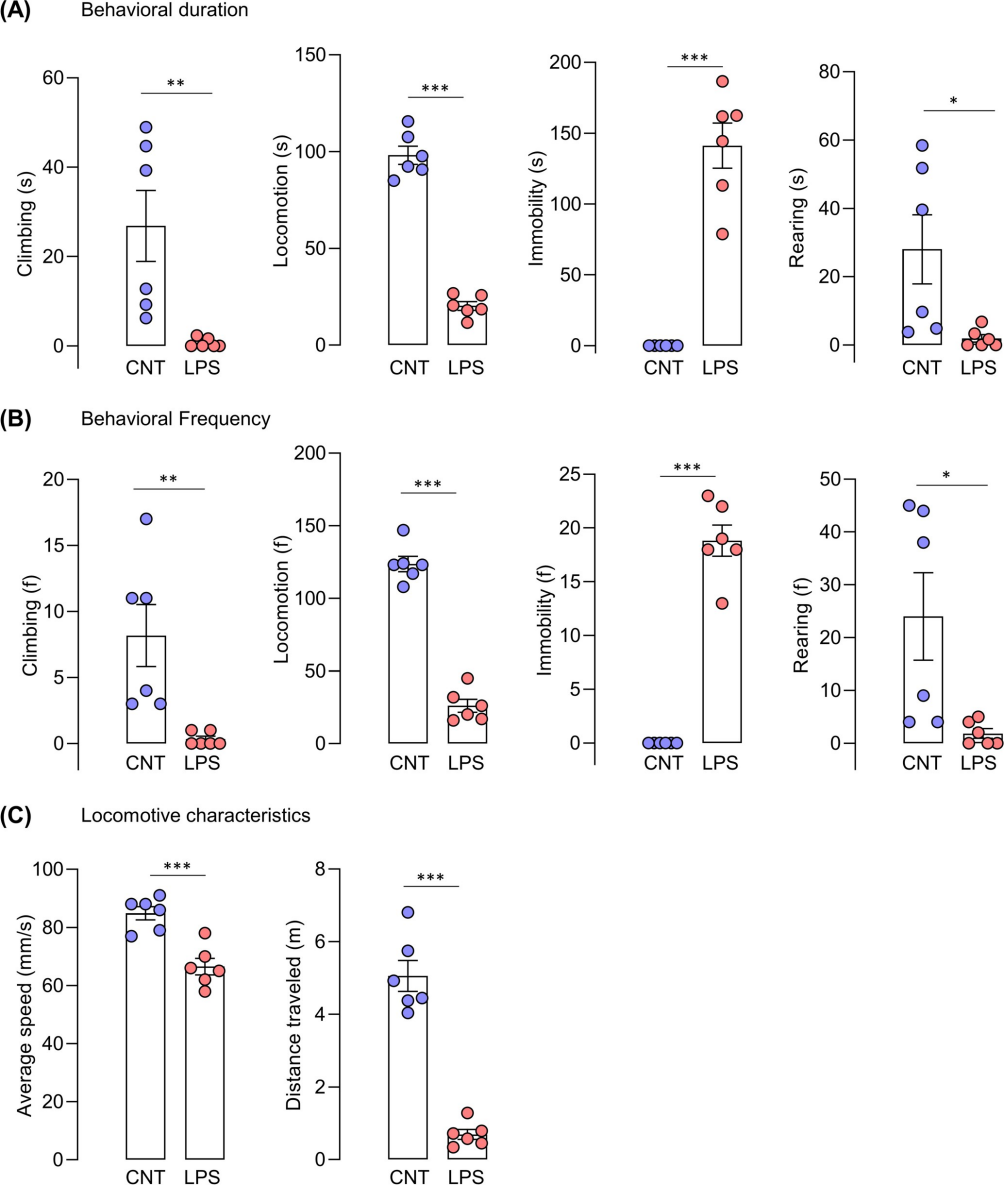
Home-cage behaviors of LPS-induced mice in 5-min period (exploratory behaviors). (A) Behavioral duration, (B) behavioral frequency, (C) locomotive characteristics. Unpaired t-test, N = 6 mice per group; *p < 0.05, **p < 0.01, ***p < 0.001 compared with the control group.

The percentage of behavioral composition, both duration, and frequency, was also assessed. The duration compositions of control and LPS groups were 17.55% and 0.40% in climbing, 64.14% and 12.33% in locomotion, 0.00% and 86.07% in immobility, and 18.31% and 1.19% in rearing, respectively. Mice in the vehicle and LPS groups showed a behavioral frequency of 5.24% and 0.71% in climbing, 79.36% and 55.32% in locomotion, 0.00% and 40.07% in immobility, and 15.40% and 3.90% in rearing, respectively (**Fig 4C**). In addition, the correlation matrix was used to assess the correlations within each behavioral category. Our results showed that mobile behaviors (climbing, locomotion, rearing) for both duration and frequency are positively correlated with each other, as well as distance traveled and average speed, while all of them are negatively correlated with immobility (**Fig 4D and S1 Data)**. Overall, LPS-treated mice showed significantly lesser mobile behaviors including climbing (t _(10)_ = 3.291 p = 0.0081 for climbing duration; t _(10)_ = 3.328 p = 0.0076 for climbing frequency), locomotion (t _(10)_ = 15.00 p < 0.0001 for locomotion duration; t _(10)_ = 14.05 p <0.0001 for locomotion frequency), rearing (t _(10)_ = 2.555 p = 0.0286 for rearing duration; t _(10)_ = 2.658 p = 0.024 for rearing frequency) and significantly higher immobility (t _(10)_ = 8.856 p < 0.0001 for immobility duration; t _(10)_ = 13.01 p < 0.0001 for immobility frequency) compared to that of the control group indicated by duration and frequency of behaviors (**Fig 5A and 5B**). In line with this result, other behavioral characteristics such as average speed (t _(10)_ = t = 5.057 p = 0.0005) and distance traveled (t _(10)_ = 9.771 p < 0.0001) were also reduced in the LPS group compared to that of the control group **(Fig 5C**).

### LPS-induced sickness behaviors, long-term locomotor activity in the automated home-cage monitoring

**Fig 6:**
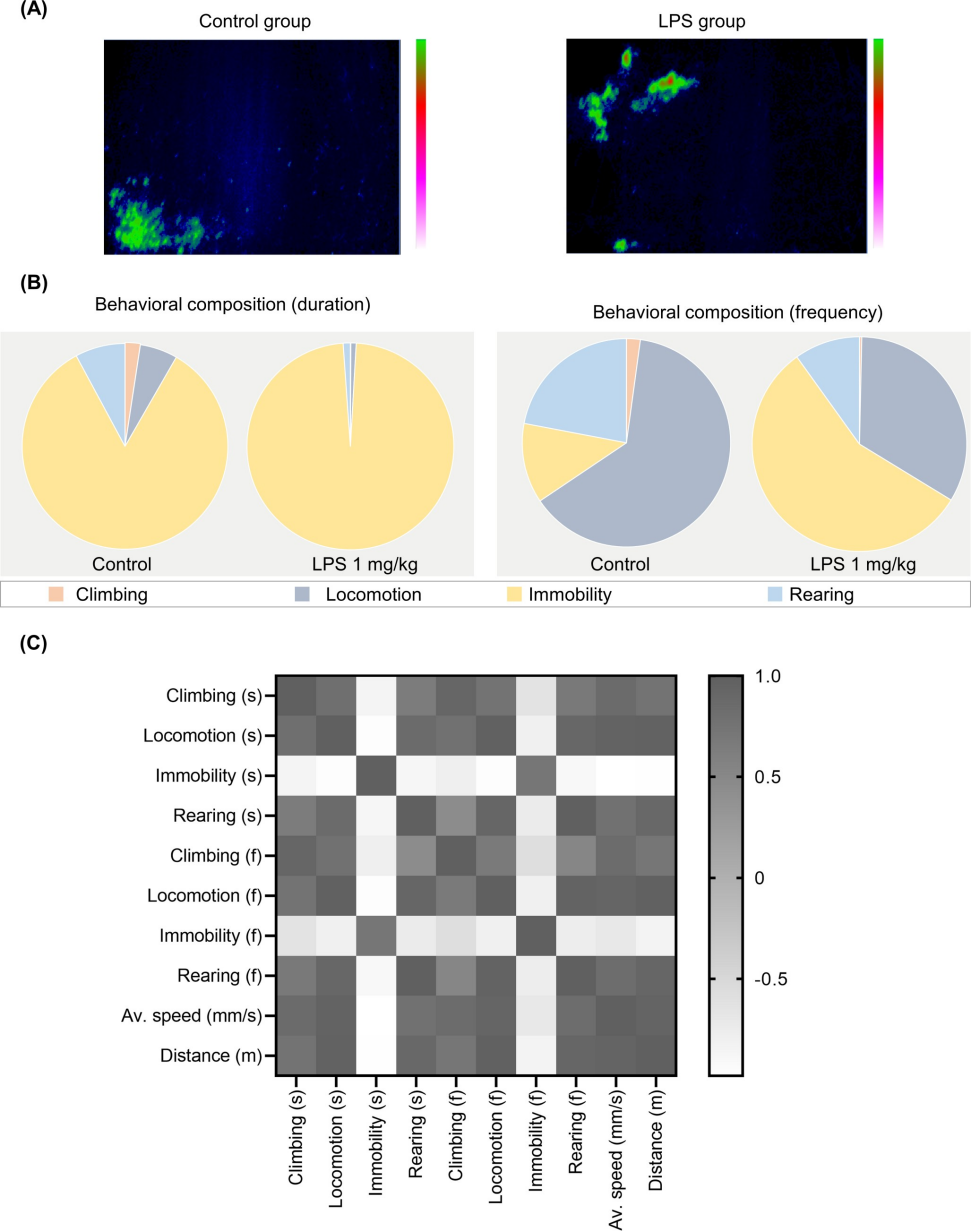
Automated home-cage behavioral phenotyping of LPS-induced mice. (A) position distribution, (B) percentage of behavioral composition (duration and frequency), derived by either duration or frequency of certain behavior/total duration or frequency of identified behaviors, (C) correlation matrix of each behavior.

To gather further information about the innate behavioral profile related to the locomotor activity associated with LPS-induced sickness behavior for a 24 h period in the light and dark conditions, mice were subjected to automated home-cage monitoring using the automated home-cage system LABORAS, while the mice had free access to food and water (**Fig 3A**). We quantified different behaviors: climbing, locomotion, immobility, rearing, average speed, and distance traveled along with the body weight change, food, and water intake (**Fig 3B**). In this test, behavioral tests were performed between 18.00 and 17.59 h (24 h period). Generally, within 1 h, mice explored the entire cage as a response to a novel environment. To effectively assess the position of the mouse in the cage, the automated home-cage generated a position distribution. As shown in **Fig 6A**, mice in both groups mostly preferred to stay at one corner of the cage. The time spent in each behavioral category, for the vehicle and LPS groups were 2.40% and 0.07% in climbing, 5.92% and 0.85% in locomotion, 83.83% and 97.97% in immobility, and 7.86% and 1.12% in rearing, respectively. In addition, the frequency of behavioral events was also quantified. Mice in the vehicle and LPS groups displayed behavioral composition at 2.15% and 0.35% in climbing, 63.42% and 33.37% in locomotion, 12.42% and 56.37% in immobility, and 22.01% and 9.91% rearing, respectively (**Fig 6B**). To determine the relationship between the measured behaviors, a correlation matrix was constructed from the data of the vehicle and LPS groups. The results showed that climbing, locomotion, and rearing behaviors (duration and frequency) positively correlated with each other, as well as the maximum speed, average speed, and distance traveled, but negatively correlated with immobility (duration and frequency) **(Fig 6C and S2 Data)**.

**Fig 7:**
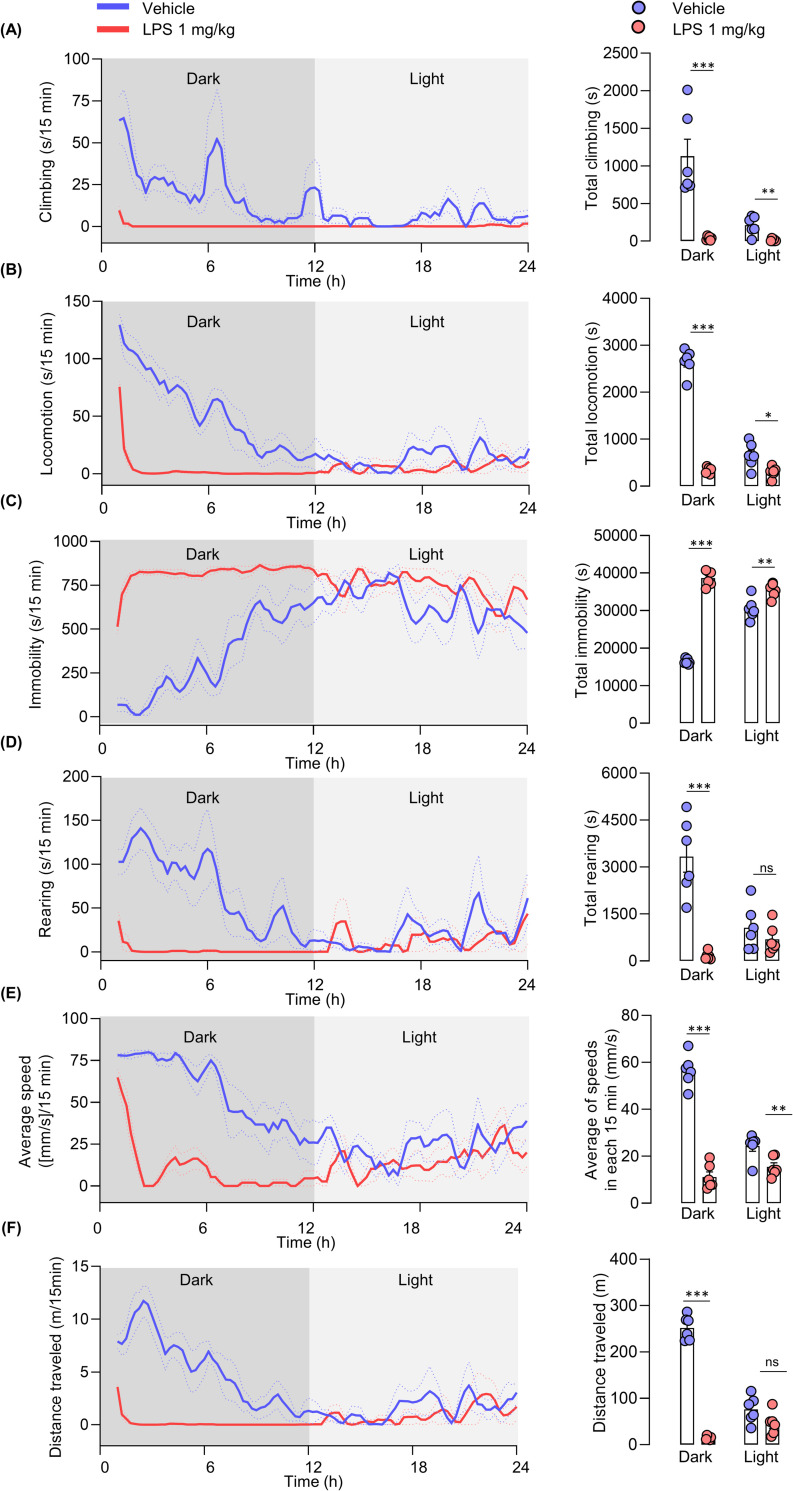
**Effect of LPS on the duration of home-cage behaviors, average speed, and distance traveled throughout a 24 h period (dark and light phases).** Each line depicts the moving average of each behavior for both control and LPS groups. (A) Climbing, (B) locomotion, (C) immobility, (D) rearing, (E) average of speeds in every 15 minutes (mm/s), and (F) distance traveled (m). Unpaired t-test, N = 6 mice per group; *p < 0.05, **p < 0.01, ***p < 0.001 compared with the control group.

Altogether the LPS-induced mice demonstrated a significant decrease in duration of climbing behavior as compared to the vehicle control group during the dark phase (t _(10)_ 4.823, p = 0.0007) and the light phase (t _(10)_ = 3.955, p = 0.0027) (**Fig 7A**). In addition, the LPS group demonstrated a significant decrease in duration of locomotion and a significant increase in immobility compared to those of the vehicle control group. Since mice are nocturnal animals, a large difference in duration of locomotion (t _(10)_ = 20.23 p <0.0001) and immobility (t _(10)_ = 25.22, p <0.0001) during the dark phase and a slight difference during the light phase for both locomotion (t _(10)_ = 3.003, p = 0.01) and immobility (t _(10)_ = 3.599, p = 0.0049) was observed between the vehicle and LPS-treated groups (**Fig 7B and 7C**). Compared to the vehicle control group, the LPS group displayed a significant reduction in the duration of rearing behavior during the dark phase (t _(10)_ = 6.341, p<0.0001), but not during the light phase (**Fig 7D**). In addition, we obtained significantly lesser average speed and distance traveled in the LPS group compared to the vehicle-treated group during the dark phase (t _(10)_ = 12.94, p < 0.0001 for average speed; t _(10)_ = 22.06, p < 0.0001 for distance traveled) and light phase (t _(10)_ = 3.200 p = 0.0095 for average speed) (**Fig 7E and 7F**).

**Fig 8:**
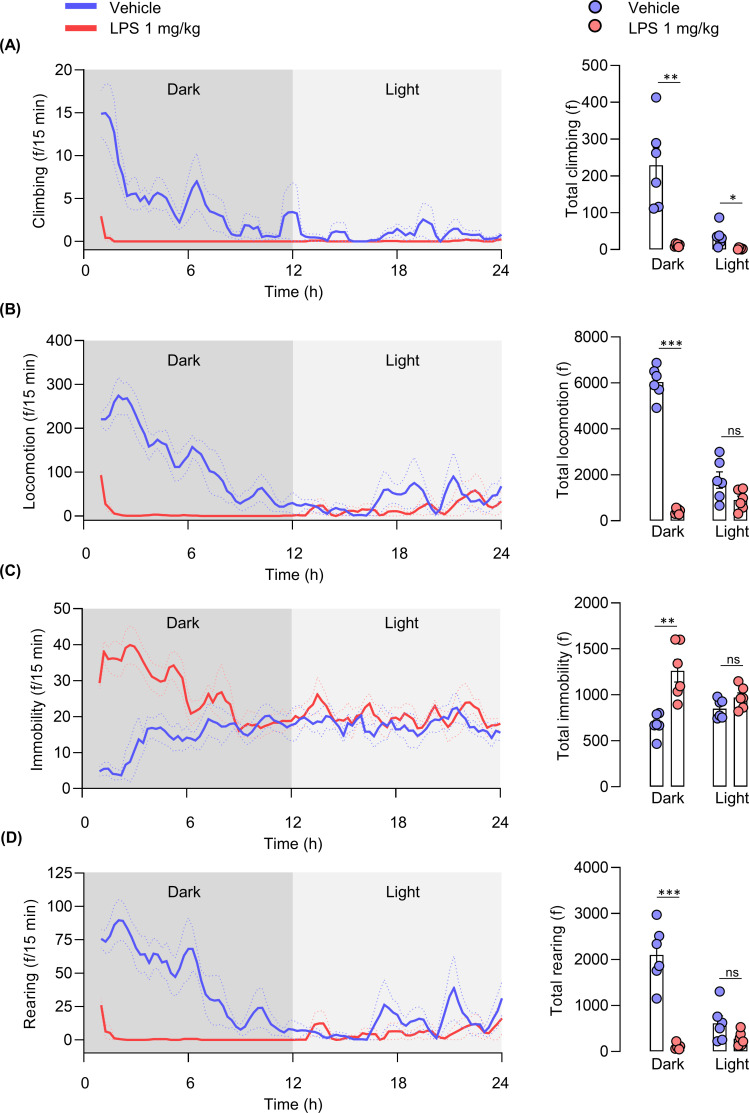
**Frequency of home-cage behaviors of LPS-induced mice throughout a 24 h period (dark and light phases).** Each line depicts the moving average of each behavior for both control and LPS groups. (A) Climbing, (B) locomotion, (C) immobility, (D) rearing. Unpaired t-test, N = 6 mice per group; *p < 0.05, **p < 0.01, ***p < 0.001 compared with the control group.

As per the behavioral frequency data, mice in the control group elicited more mobile behaviors: climbing (t _(10)_ = 4.577, p = 0.001), locomotion (t _(10)_ = 19.72, p < 0.0001) and rearing (t _(10)_ = 7.546, p < 0.0001), and less immobility (t _(10)_ = 4.417, p = 0.00130), compared to that of the LPS group during the dark phase (**Fig 8A–8D**). A significant change between LPS and vehicle-treated groups during the light phase was observed only in climbing frequency (t _(10)_ = 3.015, p = 0.013) (**Fig 8A**). Simultaneously, other behaviors showed no significant difference compared to that of the vehicle control group (**Fig 8B–8D**).

### Bodyweight changes, food, and water intake

**Fig 9:**
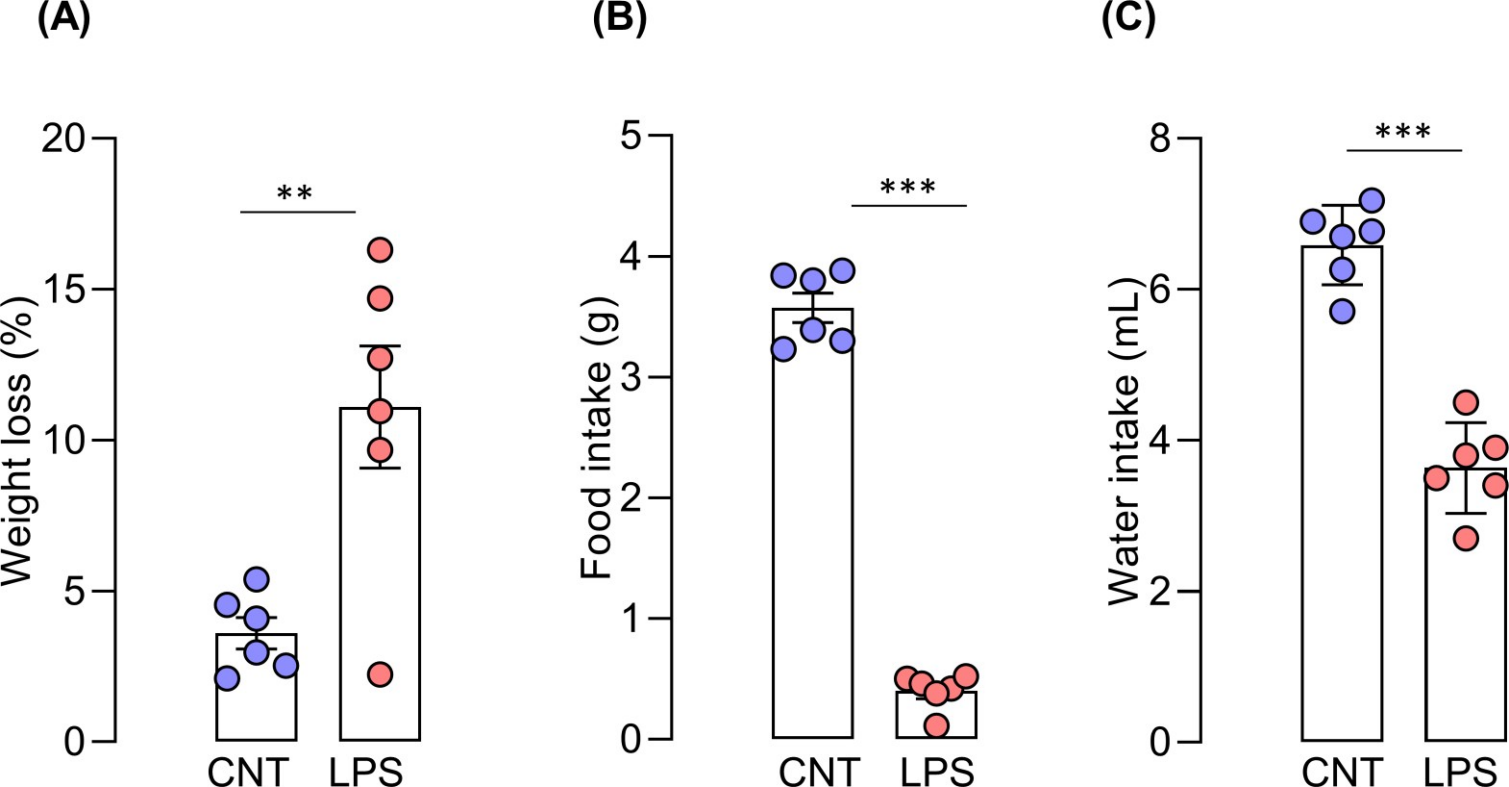
Bodyweight changes, food, and water intake. (A) Percentage of body weight changes, (B) Food and (C) water intake of mice after LPS exposure. Unpaired t-test, N = 6 mice per group; ** p < 0.01, *** p < 0.001 compared with the control group.

Mice in the vehicle control group displayed only a slight weight loss of 3.3%, whereas the LPS group displayed a more significant weight loss of 12.2% (t _(10)_ = 3.582, p = 0.005) (**Fig 9A**). In addition, the LPS group also exhibited a significant decrease in food (t _(10)_ = 23.33, p < 0.0001) and water intake (t _(10)_ = 9.092, p < 0.0001) compared to that of the vehicle control group, which displayed a percentage of reduction of 88.9 and 44.8, respectively (**Fig 9B and 9C)**.

### Pharmacological intervention using indomethacin improves sickness behaviors in LPS-induced mice

As the LPS-induced mice displayed sickness behaviors associated with immune system activation, we reasoned that indomethacin administration might improve the sickness behaviors along with the pain-like behaviors. As anticipated, the administration of indomethacin 2 h after LPS exposure improved both short-term and long-term locomotor activity.

**Fig 10:**
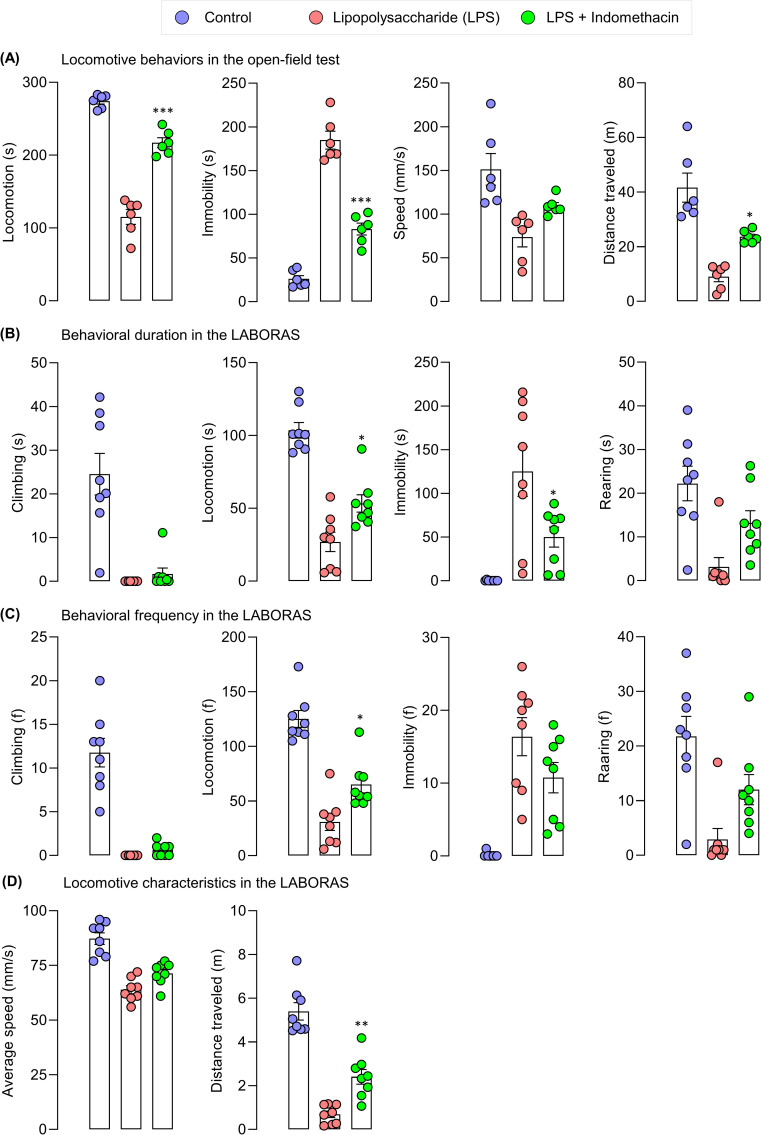
The effect of indomethacin on exploratory behaviors in LPS-induced mice. (A) Locomotive behaviors in the open-field test, (B) behavioral duration, (C) behavioral frequency, and (D) locomotive characteristics in automated home-cage LABORAS. One way ANOVA followed by Bonferroni *post hoc* test, N = 6 (open-field test) and 8 (automated home-cage LABORAS) mice per group; *p < 0.05, **p < 0.01, ***p < 0.001, LPS+indomethacin-treated group compared with the LPS-induced group.

In the short-term locomotor activity by open-field test, the administration of indomethacin increased locomotor activity (s) (F (2, 15) = 117.9, p < 0.0001), and distance traveled (m) (F (2, 15) = 24.54, p = 0.0202), decreased immobility (s) (F (2, 15) = 117.9, p < 0.0001), compared to that of the LPS group (**Fig 10A**). In exploratory behavior by LABORAS, indomethacin-treated mice demonstrated improvement in all behaviors tested, with statistically significant difference observed in locomotion (s) (F (2, 21) = 41.84, p = 0.0162), immobility (s) (F (2, 21) = 12.63, p = 0.0206), locomotion (f) (F (2, 21) = 38.39, p = 0.0144), and distance traveled (m) (F (2, 21) = 57.68, p = 0.0027) (**Fig 10B–10D**).

**Fig 11:**
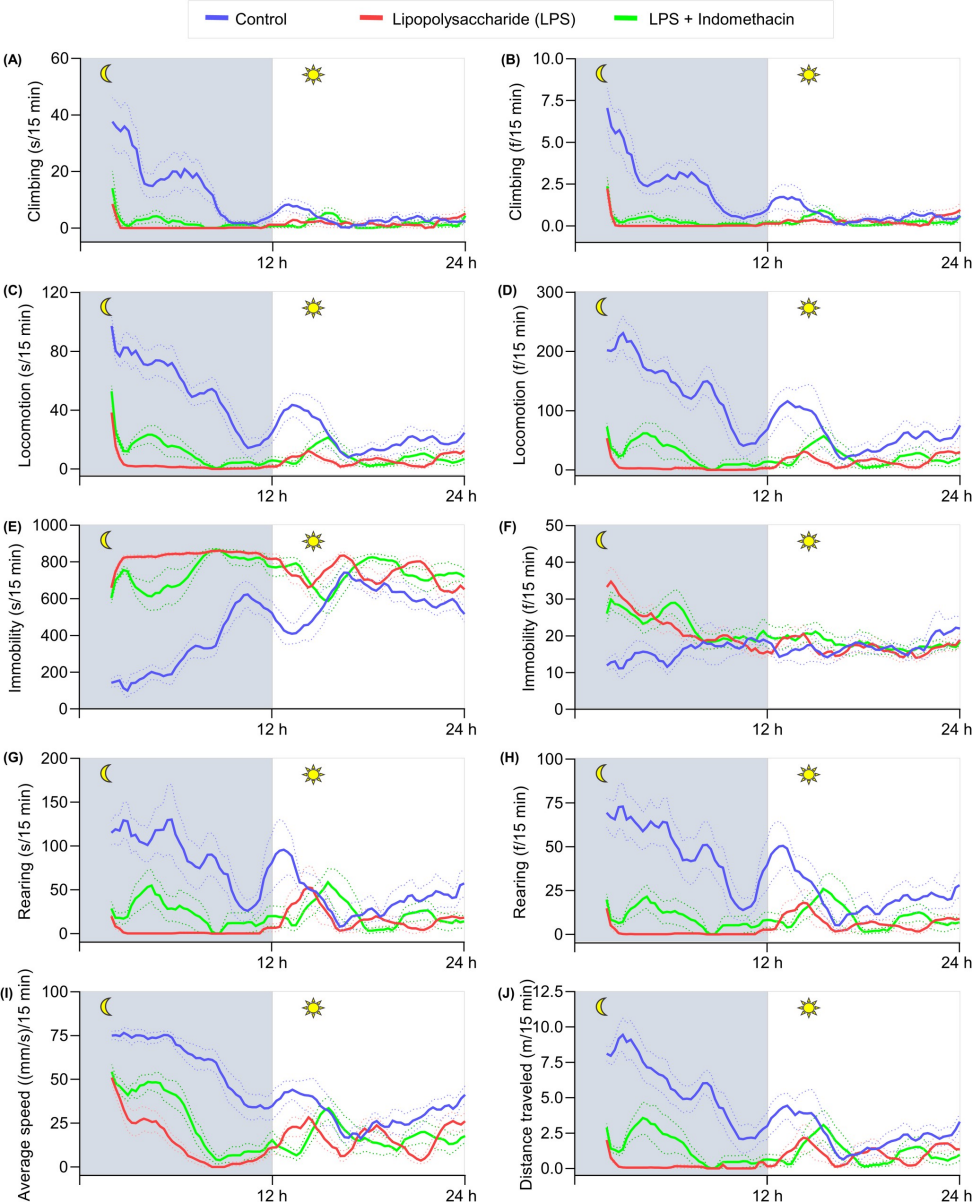
Time-series changes of home-cage behaviors in response to indomethacin treatment. Data are represented as a 2 h moving average for mean home-cage behaviors in each 15 min. Solid and dashed lines depict the mean and S.E.M of the moving average, respectively. N = 8 mice per group.

**Fig 12:**
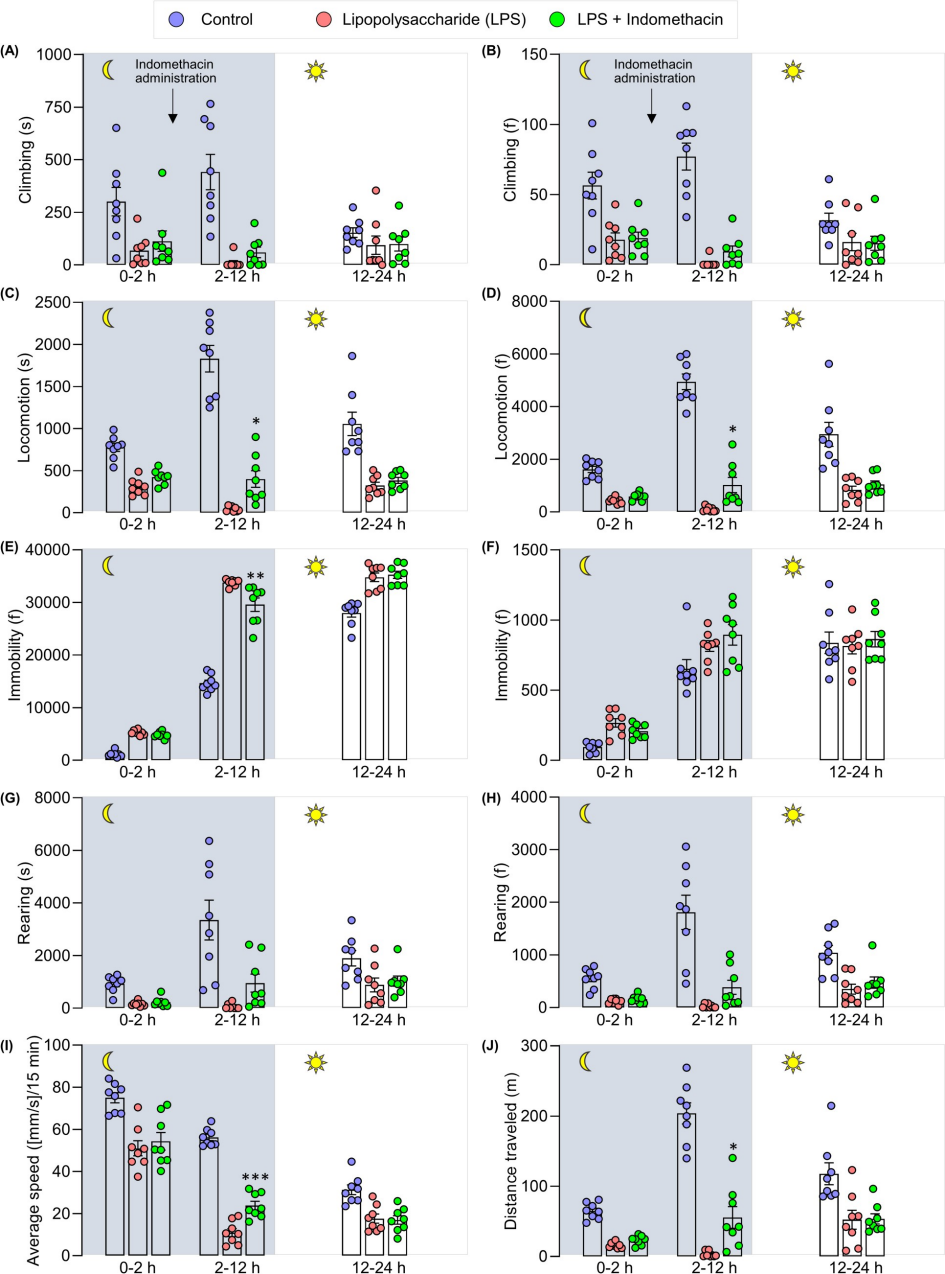
Pharmacological validation of home-cage behaviors using indomethacin in LPS-induced mice. Home-cage behaviors are divided into 3 sections: Section 0–2 h represents exploratory behaviors of mice (before indomethacin administration), section 2–12 h represents the duration of time where indomethacin is pharmacologically effective (nighttime), 12–24 h section is the duration where indomethacin is pharmacologically ineffective (daytime). N = 8 mice per group. *p < 0.05, **p < 0.01, ***p < 0.001, LPS+indomethacin-treated group compared with the LPS group. The data were analyzed by One Way ANOVA followed by Bonferroni *post hoc* test.

In the long-term locomotor activity (24 h) test, the behavioral analysis was divided into 3 sections: 0–2 h (exploratory phase), 2–12 h (nighttime), 12–24 h (daytime). The administration of indomethacin was carried out after the exploratory phase (2 h post-LPS). The time frame of home-cage behaviors is demonstrated in a line graph (**Fig 11**). As shown in **Fig 12,** during 2–12 h, the indomethacin group demonstrated improvement in all home-cage behaviors-tested with the statistical significance achieved in locomotion (s) (F (2, 21) = 106.3, p = 0.048), locomotion (f) (F (2, 21) = 117.6, p = 0.0351), immobility (s) (F (2, 21) = 151.4, p = 0.0045), average speed (mm/s) in every 15 minutes (F (2, 21) = 161.6, p = 0.0002), and distance traveled (m) (F (2, 21) = 69.30, p = 0.0219) compared to the LPS group. No substantial differences were observed in the daytime between the LPS and LPS + indomethacin groups. An improvement in weight loss (%) (F (2, 21) = 16.97, p = 0.0001), food intake (gr) (F (2, 21) = 34.71, p = 0.0169), and water intake (mL) (F (2, 21) = 31.74, p < 0.0001) were also observed (**Table 1**).

**Table 1:** Bodyweight changes, food, and water intake after administration of indomethacin.

Parameters	Vehicle control	LPS (1 mg/kg)	LPS + indomethacin (10 mg/kg)	F (DFn, DFd), p value (LPS vs indomethacin)
Weight loss (%)	5.65 ± 0.93	11.88 ± 1.05	5.47 ± 0.62***	F (2,21) = 16.97, p = 0.0001
Food intake (g)	3.75 ± 0.40	0.69 ± 0.14	1.84 ± 0.17***	F (2,21) = 34.71, p = 0.0169
Water intake (mL)	5.39 ± 0.45	0.87 ± 0.13	4.01± 0.53 ***	F (2,21) = 31.74, p < 0.0001

N = 8 mice per group.

***p < 0.001 compared with the LPS group. The data were analyzed by One Way ANOVA followed by Bonferroni *post hoc* test.

## Discussion

In the present study, we employed automated home-cage monitoring to investigate the pain-like behaviors associated with the sickness behaviors induced by LPS in mice. Our work found that mice treated with LPS exhibited impaired short-term (exploratory behaviors) and long-term locomotor activity. The short-term locomotive behavior of mice was assessed in both open-field test (conventional locomotor test) and automated LABORAS. In the open-field test, LPS-induced mice demonstrated decreased locomotor activity, which in line with the results of exploratory behavior analyzed by LABORAS. However, automated home-cage monitoring LABORAS could provide a comprehensive and detailed analysis of behaviors at each time point. In addition, to assess long-term locomotor activity in both the dark and light phases, we utilized automated home-cage monitoring LABORAS. The results demonstrated that in both phases, the mobile behaviors (climbing, locomotion, rearing), average speed, and distance traveled were significantly decreased after LPS administration, while immobility was increased. Moreover, the difference in the locomotive behaviors between the LPS-induced mice and control mice was more evident in the dark phase compared to the light phase. Further, to validate the model, indomethacin, an anti-inflammatory agent, was employed as a pharmacological intervention, which improved sickness and pain-like behaviors in both the classical locomotor test and automated home-cage monitoring.

Achieving a high level of accuracy in interpreting pain-like behaviors in rodents is the main goal when conducting behavioral tests. Previous studies have found that reflexive-pain tests have several limitations such as labor and time consuming that profoundly impact the experimental data interpretation [38,40]. Given the limitations of using reflexive pain behavioral testing, we propose locomotor activity, a non-reflexive pain behavior measured by automated home-cage monitoring as an alternative for indirect pain assessment in rodents. Locomotor activity is commonly used as a readout of behavioral pain in numerous pain models, including carrageenan-induced inflammatory pain [47], complete Freund’s adjuvant (CFA)-induced inflammatory pain [48], spinal cord injury [49], and postoperative pain [50]. In our study, we provide a detailed characterization of the locomotive behaviors of LPS-stimulated mice in the home-cage system. Compared to the open-field, the acquisition of locomotive behaviors in the automated home-cage was more comprehensive and detailed. The behaviors acquired from LABORAS, including climbing, rearing, walking, running, immobility, were captured precisely in each second making it suitable to be easily analyzed as well as visualized in an ethogram. In addition, we performed a long-term locomotor activity test by automated LABORAS home-cage in the biological diurnal rhythms of mice. These long-term behavioral measurements are lacking in the preclinical assessment of pain. Compared to the conventional open-field test, a long-term locomotive activity test for 24 hours has so far only been possible in automated home-cage monitoring. In the home-cage system, mice are kept in a home-like environment, with less stress and free access to food and water. A less stressful environment may improve the translational value of the preclinical study, where stress can cause analgesia or hyperalgesia [55,56]. In the present study, we found that the administration of LPS to mice reduced the short-term locomotor activity in both the open-field and the automated home-cage monitoring system. In the home-cage, LPS decreased mobile behaviors (climbing, locomotion, rearing), average speed, distance traveled, food and water intake, and increased immobility, indicating the physical disability of mice in short-and long-term locomotive assessments. Moreover, the decreased locomotive activity found in our study is consistent with the findings of the previous studies [57–59].

Automated home-cage monitoring is a powerful tool to characterize the behavioral pain in rodents and has been characterized in several animal models of pain such as spared nerve-injured (SNI)-mice [60], post-operative mice [61], complete Freund’s adjuvant (CFA)-induced mice [62], mice with collagen-induced arthritis [63,64], mice with osteoarthritis induced by transection of the medial meniscotibial ligament [65], and rats with spontaneous trigeminal allodynia (STA) [66]. In the SNI-mouse model, an animal model of neuropathic pain demonstrated a trend of reduced mobile behaviors (climbing) [60]. Moreover, CFA-induced mice demonstrated reduced climbing and locomotive behaviors, distance traveled and average speed compared to the control group [62]. In mice with experimental osteoarthritis (OA), significant changes were observed in climbing, locomotion, and immobility between OA-mice and the sham mice [65]. In mice with collagen‐induced arthritis (CIA), significant behavioral changes were observed between the arthritis and control non-arthritis mice as indicated by decreased time spent in climbing, locomotion and grooming, and increased immobility time [63]. Despite all the significant changes demonstrated in the aforementioned models, rats with spontaneous trigeminal allodynia (STA) and partial meniscectomy (PMX)-operated animals did not display clear behavioral changes compared to controls [64,66]. In some instances, analysis of these behaviors enables differentiation between normal mice and mice with pain, which also has been elucidated in our study. Such a tool enables the experimenter to take a closer look into the animals’ physical activity (locomotor activity) associated with pain. Automated home-cage monitoring LABORAS not only helps automate tracking, but also captures behaviors precisely in seconds to minutes, hours, and days that allows unbiased observations leading to precise quantification and identification of behavioral pain [40,52]. In this approach, the characterization of mobile behaviors (climbing, rearing, and locomotion) and immobility could provide important predictive and representative information of behavioral signs of LPS-induced sickness behaviors along with pain-like behaviors in rodents.

The present study further demonstrated pharmacological characterization of the home-cage behaviors using indomethacin, a gold-standard agent for treating inflammation, and often used as a comparator of analgesic candidate drugs. Despite its action as a COX-2 inhibitor, indomethacin blocks several inflammatory mediators, such as cytokines, chemokines, and growth factors [67–69]. In our study, indomethacin administered to LPS-treated mice was efficacious in all tests, which showed a significant increase in locomotor activity compared to the LPS alone group. These results are in line with previous studies which showed the efficacy of indomethacin [51]. In automated home-cage monitoring, indomethacin also increased the time and frequency of mobile behaviors and decreased immobility. The data of home-cage behaviors provide information that indomethacin, an anti-inflammatory drug improved behavioral signs of sickness behaviors.

The innate behaviors of LPS-induce mice in automated home-cage monitoring should be carefully interpreted as behavioral pain since LPS was administered intraperitoneally. Although intraperitoneal administration of LPS has been devised in several pain models, such as sepsis-associated pain, musculoskeletal pain, and visceral pain [11,18–20,35], the intraperitoneal administration of LPS can cause a systemic immune response leading to the development of sickness behaviors. Sickness behaviors produced by intraperitoneal administration of LPS include fatigue, depressive-like behavior, feeding behavior, anxiolytic-like behavior, changes in sleep-wake and feeding behaviors, weight loss, fever, and pain-like behaviors [8–13]. Hence, home-cage behaviors in LPS-induced mice not only represent the pain-like behaviors but also possibly other parameters, such as fatigue, changes in diurnal rhythms (sleep pattern), and motivational changes. Therefore, in order to represent specific behaviors in sickness behaviors induced by the LPS, automated home-cage monitoring should be combined with other conventional behavioral assessments. For example, in one study, automated home-cage monitoring LABORAS was combined with other conventional tests (open-field test, elevated plus maze test, light-dark test, and fear conditioning test) to measure hypoactivity and anxiety-like behavior [70]. In the assessment of sleep/wake states of mice, LABORAS and SmartCage™ automated home-cage monitoring were combined with electroencephalography (EEG) and electromyography (EMG) to capture sleep pattern precisely [71,72]. Therefore, to objectively capture pain-like behaviors in mice intraperitoneally administered with LPS, the automated home-cage behaviors should stand with reflexive pain behaviors and other non-reflexive pain behaviors. Moreover, the candidate analgesic drugs should not show any effect on locomotor activity. In some instances, analgesic agents could either enhance or decrease locomotor activity. Morphine is an opioid analgesic drug that enhances locomotor activity in rodents [73]. On the other hand, gabapentinoids such as gabapentin and pregabalin could significantly decrease locomotor activity [74]. Hence, it is recommended to evaluate the effects of potential analgesic drug candidates on motor coordination along with the locomotive behaviors to objectively interpret the home-cage behavioral data obtained in automated home-cage monitoring. The lack of data for pharmacological intervention of other inflammatory or analgesic drugs is a limitation of the present study. Given the fact that examining several drugs with distinct mechanisms of action will be beneficial for the model validation. Another limitation of the present study is that we did not experiment on female mice, instead this study focused only on male mice. A previous study reported gender-based differential response to LPS challenge [75]. The use of both sexes in an experimental design will improve the translational value of the behavioral test in animals to the clinical setting. Therefore, future studies are required to incorporate all these aspects in the experimental design.

In conclusion, automated home-cage monitoring LABORAS utilized in our study has a promising ability to unravel the sickness behaviors and give a better definition of pain-like behaviors in mice. The use of such assessments further opens new doors to pharmacological research in inflammation and pain and holds promise for pharmacological screening of potential anti-inflammatory and analgesic drug candidates. While home-cage behaviors constitute only a limited aspect of pain behaviors, a combination of reflexive and non-reflexive approaches presents an excellent opportunity to create a more holistic picture of pain behaviors. Our results establish a potential framework for quantitative and objective assessments of sickness behaviors along with pain-like behaviors in LPS-induced inflammatory mice.

## Supporting information

S1 Data Data of correlation matrix for short-term locomotor activity. (XLSX)

S2 Data Data of correlation matrix for long-term locomotor activity. (XLSX)
